# Provision of Hospital Price Information After Increases in Financial Penalties for Failure to Comply With a US Federal Hospital Price Transparency Rule

**DOI:** 10.1001/jamanetworkopen.2023.20694

**Published:** 2023-06-28

**Authors:** Edward Kong, Yunan Ji

**Affiliations:** 1 Harvard Medical School, Harvard University, Boston, Massachusetts; 2 McDonough School of Business, Georgetown University, Washington, DC

## Abstract

**Importance:**

Price transparency regulations aim to help patients make informed decisions about medical care, but enforcing these rules is a policy challenge. There may be an association between financial penalties and hospital compliance for enforcing price transparency regulations.

**Objective:**

To evaluate the association between financial penalties and acute care hospital compliance with the 2021 Centers for Medicare & Medicaid Services (CMS) Price Transparency Rule.

**Design, Setting, and Participants:**

This cohort study uses an instrumental variable design to evaluate the responses of 4377 acute care hospitals in the US operating in 2021 and 2022 to changes in financial penalties in the context of a federal rule requiring hospitals to disclose privately negotiated prices.

**Exposure:**

Changes in noncompliance penalties between 2021 and 2022 based on a nonlinear function of bed counts.

**Main Outcomes and Measures:**

Whether hospitals publicly posted a machine-readable file with private, payer-specific negotiated prices at the service-code level. Negative controls were used to address confounding.

**Results:**

The final sample included 4377 hospitals. Compliance increased from 70.4% (n = 3082) in 2021 to 87.7% (n = 3841) in 2022, with 90.2% of hospitals (n = 3948) reporting prices in at least 1 year. Noncompliance penalties increased from $109 500/y in 2021 to a mean (SD) of $510 976 ($534 149)/y in 2022. Penalties in 2022 were substantial, averaging 0.49% of total hospital revenue, 0.53% of total hospital costs, and 1.3% of total employee wages. Compliance increases were significantly positively correlated with penalty increases: a $500 000 increase in penalty was associated with a 2.9–percentage point (95% CI, 1.7-4.2 percentage points; *P* < .001) increase in compliance. Results were robust to controlling for observable hospital characteristics. No associations were found for preperiod (2021) compliance or ranges of bed counts where penalties do not vary.

**Conclusions and Relevance:**

In this cohort study of 4377 hospitals, compliance with the CMS Price Transparency Rule was associated with increased financial penalties. These findings are relevant for the enforcement of other regulations designed to promote transparency in health care.

## Key Points

**Question:**

Are potential financial penalties associated with hospital compliance with federal price transparency regulations?

**Findings:**

In this cohort study of 4377 acute care hospitals, compliance with the 2021 Centers for Medicare & Medicaid Services Price Transparency Rule increased from 70.4% in 2021 to 87.7% in 2022. Increases in compliance were significantly positively correlated with penalty size, which changed from a flat rate to a function of hospital bed counts.

**Meaning:**

Hospital compliance with federal price transparency regulations is high and increasing; this study suggests that financial penalties may be a useful policy enforcement mechanism in health care.

## Teaser

This cohort study of more than 4000 US acute care hospitals evaluates the association between financial penalties and hospital compliance with the 2021 Centers for Medicare & Medicaid Services Price Transparency Rule.

## Introduction

Price transparency regulations aim to help patients make informed decisions about medical care, but enforcing these rules is a policy challenge. Effective January 1, 2022, the Centers for Medicare & Medicaid Services (CMS) increased financial penalties for noncompliance, from $300/d per hospital (for all hospitals regardless of hospital size) in 2021 to a sliding scale between $300/d and $5500/d in 2022.^1^

We report novel compliance data over 2 years (2021 and 2022) from almost all acute care hospitals in the US and assess whether increased financial penalties were useful for increasing hospital compliance with the CMS Price Transparency Rule. We report new compliance rates and evaluate why compliance estimates vary widely in the literature.^2,3,4,5,6,7,8,9,10,11^ Leveraging our large sample size and an instrumental variable research design with negative controls^12^ (sometimes referred to as *placebo tests*), we asked whether increases in compliance were associated with increases in penalties across hospitals. We hypothesized that hospitals facing greater increases in penalties between 2021 and 2022 had higher increases in compliance.

## Methods

### Study Sample

Following our prior work,^11^ our starting data set included 4689 acute care hospitals in the 2017 American Hospital Association survey. We limited the data set to hospitals with hospital-level characteristics available in the CMS Provider of Services File,^13^ additional covariates from the Dartmouth Atlas of Health Care,^14^ and bed counts from the 2018 CMS Hospital Cost Reports^15^ (the exact data used by CMS to compute penalties in 2022). We additionally excluded hospitals that closed prior to the end of 2022 and integrated health systems that do not take private insurance. See eFigure 1 in Supplement 1 for details on sample flow. The Harvard University Institutional Review Board waived approval because the study was deemed as not constituting human participant research. This study followed the Strengthening the Reporting of Observational Studies in Epidemiology (STROBE) reporting guideline.

### Primary Outcome

We classified a hospital as compliant in 2022 if it publicly posted a machine-readable file with private, payer-specific negotiated prices at the service-code level. In contrast to some of the existing literature, we did not impose additional requirements in our compliance definition (eg, presence of “shoppable service tools” or correct file-naming convention). To establish final compliance rates for each year, we checked the website for each hospital in December 2021 and December 2022 to confirm whether a file was posted by the end of the year. To ensure data accuracy, the compliance status of each hospital was independently verified by 2 separate team members including at least 1 author.

We conducted our analyses at the hospital level (the level at which penalties are defined). Our primary outcome variable was the hospital-level change in compliance from 2021 to 2022, using values of −1 (when a previously compliant hospital became noncompliant) to 1 (for newly compliant hospitals).

### Research Design

We leveraged a natural experiment based on differential changes to the noncompliance penalty. In 2021, financial penalties were $300/d for all hospitals, regardless of bed count. On January 1, 2022, financial penalties were increased to $10 per bed per day, with a minimum of $300/d for hospitals with no more than 30 beds and a maximum of $5500 for hospitals with 550 or more beds. This represented a range of increases between 0% and 1733% of the original penalty amounts. These theoretical penalties were made credible by CMS enforcement actions; in June 2022, 2 hospitals were fined a total of $1.2 million.^16^ Because these were the only hospitals actually levied penalties, our results should be interpreted in terms of potential or threatened penalties. As our data collection efforts span the periods before and after these policy changes, we were positioned to study the association of these penalty increases with compliance.

Our research design had several features that helped to address potential confounding. First, our multiyear data set allowed us to examine changes in compliance, to eliminate spurious correlations resulting from observed and unobserved hospital characteristics that have constant associations over time (for example, a tendency for larger hospitals to have higher baseline levels of compliance). Second, bed count was not associated with penalties for hospitals with fewer than 31 beds or more than 550 beds, giving us a natural negative control (aka placebo test). Third, we used a second negative control by correlating bed count with compliance in 2021, prior to the penalty change. These 2 tests helped us rule out confounding based on unobservable characteristics associated with bed count.

We used an instrumental variables design to quantify the association between compliance and financial penalties. Our instruments were the bed count categories defined by the 2 kinks in the 2022 penalty schedule (≤30, 31-550, >550) and the interaction with the number of inpatient beds. We used the statutory relationship between penalty amounts and bed count as our first stage (as a result, the relevance condition was mechanically satisfied; see the eAppendix in Supplement 1 for more details on the instrumental variables design). Regarding the instrumental variables exclusion restriction, one concern was that bed count was likely to be correlated with unobserved hospital characteristics that were associated with compliance (for instance, better information technology resources at larger hospitals). To address this possibility, we leveraged sharp changes in the slope of the penalty schedule at 30 and 550 beds; we assumed that differences in bed count near these thresholds were associated with changes in compliance only through their association with penalty changes. This assumption allowed us to control for bed count linearly in the regression. Using the change in compliance as our outcome variable also helped to exclude any fixed differences in compliance between smaller and larger hospitals. For example, while we verified that larger hospitals had higher initial compliance in 2021 (potentially due to better data infrastructure), our estimates would only be biased to the extent that larger hospitals accelerated compliance between 2021 and 2022.

Because penalty changes increased between 31 and 550 beds, we expected changes in compliance to have a positive correlation with bed counts in this range. Because penalties did not vary with bed counts of up to 30 or above 550, we expected a flatter association between changes in compliance and bed count in these ranges. The nonlinearities in the penalty schedule were helpful for ruling out biases associated with hospital size: such bias would need to follow the same nonlinear pattern in the penalty schedule (ie, association with increasing compliance only for hospitals between 31 and 550 beds, with flat compliance rates for hospitals with up to 30 or more than 550 beds). We compared our instrumental variables estimates with their ordinary least-squares analogs of regressing change in compliance on change in penalties. Last, we summarized compliance estimates from the literature to show how compliance estimates have changed over time, holding the definition of compliance constant.

### Statistical Analysis

For estimation, we used 2-stage least-squares to estimate instrumental variables specifications and ordinary least-squares to estimate other specifications. We conducted statistical tests using robust SEs and a 2-sided significance level of *P* < .05. All statistical analyses were conducted in Stata, version 17 (StataCorp LLC).

## Results

Between 2021 and 2022, overall compliance rates for the 4377 hospitals in our sample increased by 17.3 percentage points, from 70.4% (n = 3082) in 2021 to 87.7% (n = 3841) in 2022, with 90.2% (n = 3948) of hospitals reporting prices in at least 1 year. Put differently, noncompliance rates decreased by over half, from 29.6% (n = 1295) to 12.3% (n = 536).

As shown in Table 1, the annual financial penalty was $109 500 in 2021 for every hospital; this reflects the previous penalty schedule, which set a constant penalty across all hospitals ($300/d × 365 days). The mean (SD) penalty was $510 976 ($534 149) in 2022, with significant variation across hospitals due to the new sliding penalty scale. Penalty increases were larger among newly compliant hospitals and always-compliant hospitals. Penalty amounts were significant: the relative magnitude of penalties averaged 0.49% of total hospital revenue, 0.53% of total hospital costs, and 1.3% of total employee wages. For comparison, penalties in the Hospital Readmissions Reduction Program (HRRP) were about 0.36% of total hospital revenues. Our process for calculating this statistic is as follows: (1) HRRP caps penalties at 3% of Medicare fee-for-service (FFS) payments^17^; (2) Medicare FFS payments totaled $119 billion in 2017^18^; and (3) total hospital revenues in 2017 included $498 billion in inpatient payments and $472 billion in outpatient payments.^19^ Hence, Medicare FFS accounts for 119/(498 + 472) = 12% of total hospital revenues. This implies that the maximum HRRP penalties are 3% × 0.12 = 0.36%, which is somewhat lower than the magnitude of noncompliance with hospital price transparency.

**Table 1.** Hospital Characteristics as a Function of Compliance^a^

Characteristic	Compliance group, mean (SD) value				
	All	1 (Never compliant)	2 (Newly noncompliant)	3 (Newly compliant)	4 (Always compliant)
No. hospitals	4377	429	107	866	2975
% of Hospitals	100	10	2	20	68
2021 Annual penalty, $	109 500 (0)	109 500 (0)	109 500 (0)	109 500 (0)	109 500 (0)
2022 Annual penalty, $	510 976 (534 149)	318 171 (414 821)	320 757 (331 390)	568 355 (564 377)	528 918 (539 028)
2021-2022 Penalty difference, $	401 476 (534 149)	208 671 (414 821)	211 257 (331 390)	458 855 (564 377)	419 418 (539 028)
Total revenue, $	217 494 045 (386 743 609)	124 515 258 (264 040 043)	88 363 214 (115 981 511)	257 952 067 (422 947 551)	223 566 643 (393 732 989)
2022 Penalty share of revenue, %	0.49 (0.56)	0.77 (0.84)	0.60 (0.44)	0.45 (0.71)	0.46 (0.44)
Total income, $	16 141 150 (66 799 563)	6 195 501 (52 385 384)	4 965 193 (15 251 309)	18 841 808 (60 536 221)	17 191 147 (71 236 596)
HHI market concentration^b^	1457 (1131)	1347 (983)	1529 (1342)	1417 (1066)	1483 (1160)
Market share, %	7.0 (10.8)	4.0 (6.9)	5.1 (9.5)	7.2 (10.5)	7.4 (11.3)
No. of beds	147 (191)	86 (135)	85 (92)	166 (203)	153 (195)
Multihospital system, %	64 (48)	34 (47)	55 (50)	68 (47)	68 (47)
Private for profit, %	17 (38)	18 (38)	32 (47)	8 (27)	19 (40)
Private nonprofit, %	61 (49)	45 (50)	49 (50)	74 (44)	59 (49)
Government, %	22 (41)	37 (48)	20 (40)	18 (39)	21 (41)
Teaching hospital, %	5 (22)	2 (14)	1 (10)	5 (22)	6 (23)
Critical access hospital, %	30 (73)	44 (50)	36 (48)	27 (44)	28 (45)
No ICU, %	73 (44)	50 (50)	70 (46)	77 (42)	75 (43)

Abbreviations: HHI, Herfindahl-Hirschman Index; ICU, intensive care unit.

^a^ Hospitals are classified as never compliant if they were noncompliant in 2021 and 2022, newly noncompliant if they were compliant in 2021 but noncompliant in 2022, newly compliant if they were noncompliant in 2021 but compliant in 2022, and always compliant if they were compliant in both years.

^b^ Index that ranges from 0 (perfectly competitive) to 10 000 (monopoly).

**Figure 1.**
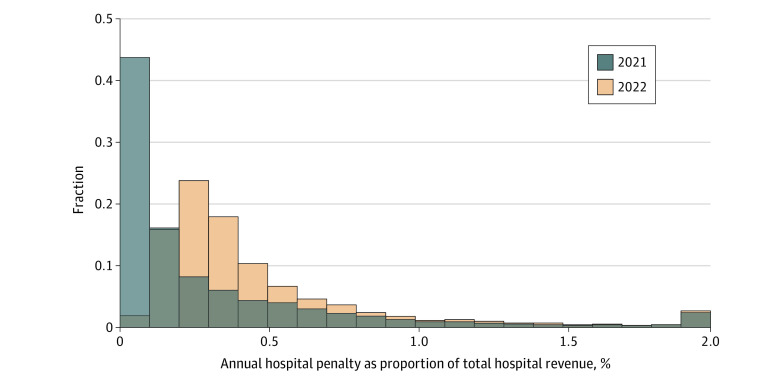
Penalties as a Percentage of Annual Hospital Revenues Histogram compares financial penalties before vs after penalties were increased in 2022. Penalties are expressed as share of annual hospital revenues in the 2018 Centers for Medicare & Medicaid Services Hospital Cost Reports. Final bin contains penalty shares of 2% or more of total annual hospital revenue.

Figure 1 shows the distribution of financial penalties as a percentage of hospital revenues. Penalties varied significantly across hospitals and could be a significant share of hospital revenues. The 2022 penalty increase was associated with a rightward shift in the relative penalty distribution. eFigure 2 in Supplement 1 reports the change in penalties in dollar amounts. eFigure 3 and eFigure 4 in Supplement 1 report the penalties as a percentage of hospital revenue, and the change in penalties as a percentage of hospital revenues, respectively, separately by bed group.

**Figure 2.**
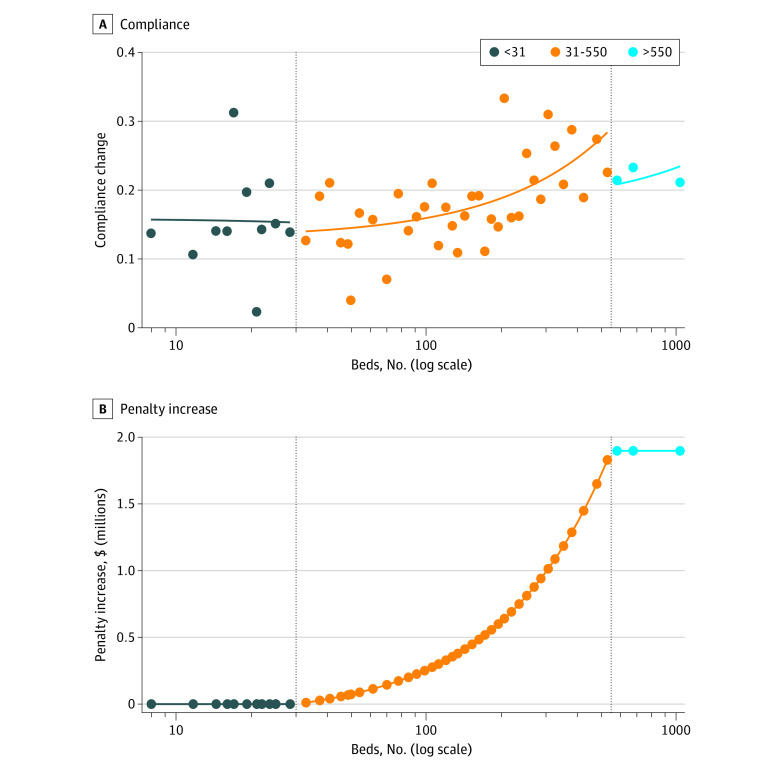
Change in Compliance vs Change in Financial Penalty (2021-2022) A, Changes in compliance share. B, Differences in penalty increases. Changes for all 4377 hospitals as a function of hospital bed count are shown (4377 hospitals binned into 50 separate bins with a median bin size of 77 hospitals; 1540 hospitals have ≤30 beds; 2651 have between 31 and 550 beds; and 186 hospitals have >550 beds). These groups represent 35%, 61%, and 4% of the data, respectively.

Figure 2 shows that the association between compliance changes and bed count closely follow the association between penalties and bed count (and is statistically significant). The figure verified our first negative control: the association between compliance and bed count was flat and not statistically significant for bed counts of up to 30 and over 550 (these negative control groups account for 35% and 4% of hospitals, respectively). This result is robust to using a linear scale for bed count (eFigure 5 in Supplement 1).

eFigure 6 in Supplement 1 verifies our second negative control: bed counts between 31 and 550 were not associated with compliance rates in 2021, before the penalty change was announced. If the patterns in Figure 1 were due to a confounding variable correlated with bed counts, we would have expected to see a similar (but spurious) correlation between bed count and compliance rates in 2021.

Table 2 shows results from our primary instrumental variables specification. We found that a $500 000 increase in penalty (approximately equal to a 1-SD increase in the 2022 penalty) was associated with a 2.9–percentage point (95% CI, 1.7-4.2 percentage points; *P* < .001) increase in compliance. The mean (SD) increase in penalty of $401 476 ($534 149) explained 13% of the increase in compliance between 2021 and 2022. Ordinary least-squares regressions gave similar, somewhat smaller associations. Our estimates were robust to controlling for bed count, limiting the sample to hospitals with 31 to 550 beds, or including additional controls for the Herfindahl-Hirschman Index, market share, hospital referral region fixed effects, whether the hospital is part of a system, hospital ownership type (nonprofit, for profit, or government), teaching hospital status, critical access hospital status, and whether the hospital has an intensive care unit (specifications with additional controls not shown). First stage estimates are reported in the eTable in Supplement 1.

**Table 2.** Association Between Penalties and Compliance

Coefficient	Compliance change					Instrumental variable estimate for negative control, 2021 compliance
	Instrumental variable estimate	Instrumental variable estimate controlled for bed count	Instrumental variable estimate for hospitals with 30-550 beds	Ordinary least-squares estimate	Ordinary least-squares estimate controlled for bed count	
Penalty change, coefficient (SE), $ (millions)	0.059 (0.0127)^a^	0.105 (0.0422)^b^	0.079 (0.019)^a^	0.056 (0.0124)^a^	0.069 (0.0319)^b^	0.024 (0.0133)
No. of beds, coefficient (SE)	NA	−0.00013 (0.000115)	NA	NA	−0.00004 (0.000088)	NA
Intercept, coefficient (SE)	0.150 (0.00835)^a^	0.151 (0.00839)^a^	0.139 (0.0132)^a^	0.151 (0.00824)^a^	.151 (0.00832)^a^	0.694 (0.00871)
No. of observations	4377	4377	2651	4377	4377	4377

Abbreviation: NA, not applicable.

^a^ Significant at *P* < .01.

^b^ Significant at *P* < .05.

**Figure 3.**
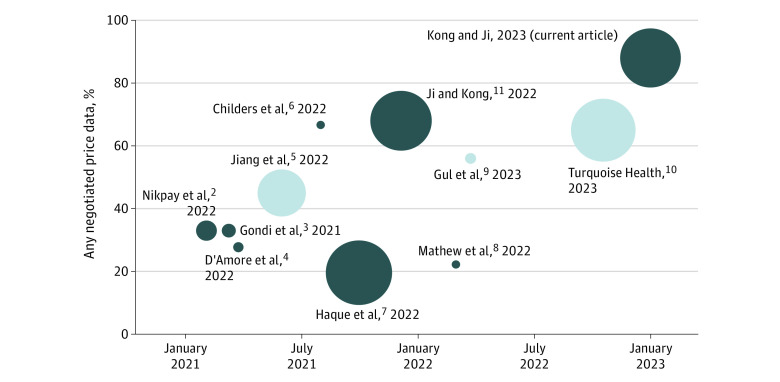
Estimates of Price Transparency Compliance in the Literature^2,3,4,5,6,7,8,9,10,11^ Compliance estimates reported in the literature by final dates of data collection are shown. All estimates are of the same definition of compliance: the percentage of sampled hospitals releasing any negotiated price data. Each circle represents a published article (except Turquoise Health,^10^ which reflects a company press release). The different sizes of circles indicate the number of hospitals included in each study. Estimates based on data from the company Turquoise Health are shown in light blue; estimates using researcher-collected data are shown in dark blue.

Figure 3^2,3,4,5,6,7,8,9,10,11^ compares our compliance rates with the literature, using a uniform definition for compliance (ie, the presence of negotiated prices). Estimated compliance rates increased over time, with some variation across different studies, some of which might be due to asymmetric measurement error (that is, true noncompliance is more difficult to verify than true compliance). Our compliance rates were in line with data collection efforts from the main commercial data provider in the industry.^10^

## Discussion

After 2 years, most US hospitals (87.7%) have made significant efforts to comply with the CMS Price Transparency Rule, as evidenced by their release of negotiated rates with private insurers. We provide the first evidence, to our knowledge, that CMS’s penalty increases for noncompliance were associated with this increase in compliance.

Other recent articles have examined compliance changes from 2021 to 2022^8,20^; however, all use much smaller samples of hospitals, do not adopt research designs that can address confounding, and draw mixed conclusions. In contrast, we maximize our statistical power by using the near universe of hospitals and are the first, to our knowledge, to use details of the CMS penalty schedule to address confounding. We show that the threat of financial penalties can induce greater levels of compliance among hospitals, in a setting where actual penalties were levied on only 2 hospitals in 1 hospital system. Our estimates suggest that increasing penalties by an another $1.4 million per hospital would increase compliance rates above 95%.

In addition to changes in compliance, other research on the Hospital Price Transparency Rule has focused largely on estimating compliance rates among smaller samples of hospitals,^2,3^ specialty hospitals,^4,6^ or use data from the commercial data company Turquoise Health,^5,9,20^ which reports a compliance rate of 65% in their data as of October 2022.^10^ Other articles have examined hospital characteristics that correlate with compliance at a given time point.^5,7^ Our own work has highlighted financial motivations such as market competition as a factor associated with compliance.^11^

Our current study adds to this literature by reporting, to our knowledge, the largest academic assessment of hospital price transparency compliance for both 2021 and 2022. Our data are unique in that they have the highest degree of completeness (ie, the share of hospitals for which we locate price data), with compliance for each hospital in each year assessed multiple times by different researchers.

Another salient feature of the literature is that compliance estimates vary greatly. Differing definitions of compliance and increases in compliance over time do not fully account for these differences. Some low compliance estimates may be due to asymmetric measurement error: it is much easier to incorrectly code a hospital as noncompliant, compared with incorrectly coding hospitals as compliant. For our definition (the presence of any private negotiated rates), compliance is easily verified (by reviewing a downloaded file), whereas noncompliance is difficult to verify (as it may involve extensive manual searches on hospital websites).

The high variation in compliance estimates is not just an academic measurement problem. Rather, this variation reflects hospitals’ incentives to “quasi-comply” by posting prices that few individuals will be able to locate but that may prevent them from being fined by the CMS. Quasi-compliance allows hospitals to minimize the costs of disclosing sensitive information while reducing their regulatory risk. Characterizing this quasi-compliance phenomenon is an important direction for future work.

Beyond the setting of hospital price transparency, our findings have policy implications for how government health care agencies can induce hospital compliance using threats of financial penalties. Historically, the CMS has used participation in Medicare as the primary mechanism for hospitals to comply with various regulations. However, there are many cases for which Medicare participation may not be the ideal enforcement mechanism. For example, since 1996, federal Health Insurance Portability and Accountability Act regulations stipulate that patients must be able to access their own medical records within 30 days, but significant noncompliance remains as of 2017.^21^ Financial penalties and incentives are widely used in other health care policies, such as the HRRP^22^; this study provides further evidence that the magnitude of penalties can matter—increasing the magnitude of potential penalties can induce larger responses.

### Limitations

This study has some limitations. The main limitation is that associations between penalties and compliance may not be causal, as penalty amounts are not randomized. Because penalties are a function of bed size, a key concern is that larger hospitals may have had more resources to comply. We addressed this possibility using 2 negative controls and demonstrated that our results are robust to controlling for beds.

Moreover, any explanation of noncompliance that invokes a lack of resources is unlikely, given that hospitals have had, at this point, 2 years to comply. A sizable fraction of small hospitals (82% of hospitals with ≤30 beds) have complied by 2022. Hospitals still not complying with the CMS policy are likely doing so for financial or strategic reasons. Thus, financial penalties and their enforcement may be necessary to induce further increases in compliance.

A second limitation is that we defined compliance based on whether hospitals posted privately negotiated prices and not other aspects of the rule, such as the consumer shoppable service tool or the exact formatting of the files posted. As a result, our estimates of compliance are higher than if all aspects of the rule were considered. Ultimately, the most relevant measure for compliance would be what hospitals believe CMS cares about when levying penalties, which is something that researchers do not observe.

## Conclusions

Overall, the results of this cohort study suggest that financial penalties may be a valuable tool for ensuring compliance with CMS policy when fines are sufficiently large, noncompliance is readily observable and well defined, and enforcement is credible. These findings are relevant for the enforcement of other regulations designed to promote transparency in health care.

## Notes

Supplement 1. **eAppendix.** Instrumental Variables Specification **eFigure 1.** Sample Definition Flow Chart **eFigure 2.** Penalty Changes From 2021 to 2022 **eFigure 3.** Penalties in 2022 as a Percentage of Hospital Revenue, by Bed Group **eFigure 4.** Change in Penalties as a Percentage of Hospital Revenue, by Bed Group **eFigure 5.** Change in Compliance as a Function of Beds (Linear X-Axis Scale) **eFigure 6.** Negative Control Based on 2021 Compliance **eTable.** First Stage and Reduced Form Regressions

Supplement 2.
Data Sharing Statement
