# Supplementary material for: Provision of Hospital Price Information After Increases in Financial Penalties for Failure to Comply With a US Federal Hospital Price Transparency Rule
Source: JAMA Netw Open. 2023 Jun 28;6(6):e2320694. doi: 10.1001/jamanetworkopen.2023.20694 (PMC10308252; doi:10.1001/jamanetworkopen.2023.20694)
Supplement: Supplement 1. — eAppendix. Instrumental Variables Specification eFigure 1. Sample Definition Flow Chart eFigure 2. Penalty Changes From 2021 to 2022 eFigure 3. Penalties in 2022 as a Percentage of Hospital Revenue, by Bed Group eFigure 4. Change in Penalties as a Percentage of Hospital Revenue, by Bed Group eFigure 5. Change in Compliance as a Function of Beds (Linear X-Axis Scale) eFigure 6. Negative Control Based on 2021 Compliance eTable. First Stage and Reduced Form Regressions [file jamanetwopen-e2320694-s001.pdf]

Supplementary Online Content

Kong E, Ji Y. Provision of hospital price information after increases in financial penalties for failure to comply with a US federal hospital price transparency rule. *JAMA Netw Open.* 2023;6(6):e2320694. doi:10.1001/jamanetworkopen.2023.20694

eAppendix. Instrumental Variables Specification
eFigure 1. Sample Definition Flow Chart
eFigure 2. Penalty Changes From 2021 to 2022
eFigure 3. Penalties in 2022 as a Percentage of Hospital Revenue, by Bed Group
eFigure 4. Change in Penalties as a Percentage of Hospital Revenue, by Bed Group
eFigure 5. Change in Compliance as a Function of Beds (Linear X-Axis Scale)
eFigure 6. Negative Control Based on 2021 Compliance
eTable. First Stage and Reduced Form Regressions

This supplementary material has been provided by the authors to give readers additional information about their work.

© 2023 Kong E et al. *JAMA Network Open.*

eAppendix. Instrumental Variables Specification

To define our instrument, we first define the following variables:

- $beds_i$ is defined as the number of inpatient beds at hospital i in the 2018 CMS cost reports data
- b_{1i} takes value 1 if the hospital has no more than 30 beds, and 0 otherwise
- b_{2i} takes value 1 if the hospital has between 31 and 550 beds (inclusive), and 0 otherwise
- b_{3i} takes value 1 if the hospital has more than 550 beds, and 0 otherwise

To capture the nonlinear penalty schedule, we define the instrument $\boldsymbol{Z_i}$ as the vector $\{b_{2i}, b_{3i}, b_{1i} \times beds_i,\ b_{2i} \times beds_i,\ b_{3i} \times beds_i\}$. Denoting the change in penalty from 2021 to 2022 faced by hospital i as $\Delta Penalty_i$, the first-stage regression is:

$$\Delta Penalty_i = \boldsymbol{Z_i \gamma} + \eta_i$$

Where η_i is the error term.

Defining $\Delta Compliance_i$ as the change in compliance from 2021 to 2022 for hospital i, we can obtain the IV estimate of the effect of $\Delta Penalty_i$ on $\Delta Compliance_i$ using two-stage least squares (TSLS):

$$\Delta Compliance_i = \beta \Delta \widetilde{Penalty_i} + \varepsilon_i$$

where $\Delta \widetilde{Penalty_i}$ is the predicted penalty from the estimated first-stage equation, ε_i is the error term, and β is the coefficient of interest describing the effect of the penalty on compliance.

© 2023 Kong E et al. *JAMA Network Open.*

eFigure 1. Sample Definition Flow Chart

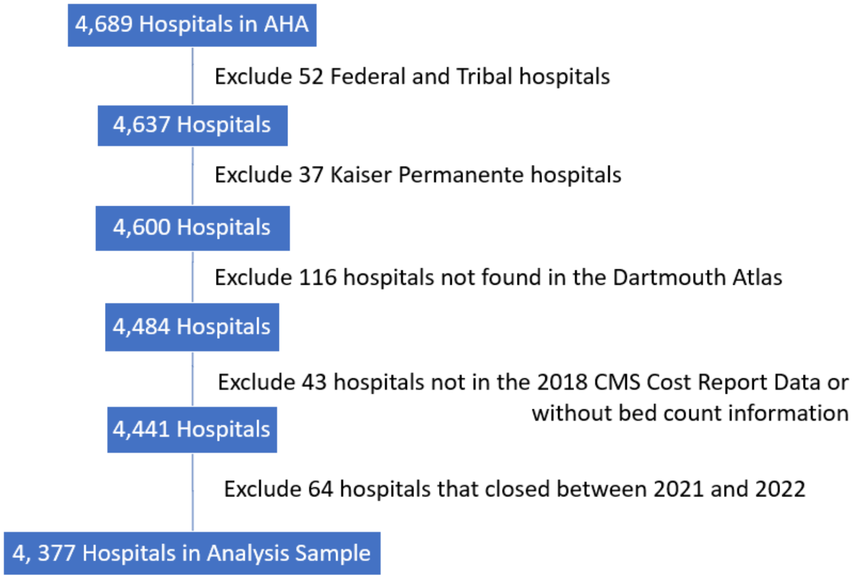

NOTES: Figure shows the step-by-step construction of the analysis sample, starting from all acute care hospitals and critical access hospitals found in the 2017 AHA data. Federal and tribal-owned hospitals (defined using the CMS Provider of Services File) are exempt from the price transparency rule (see 45 CFR 180.30).

© 2023 Kong E et al. *JAMA Network Open.*

eFigure 2. Penalty Changes From 2021 to 2022

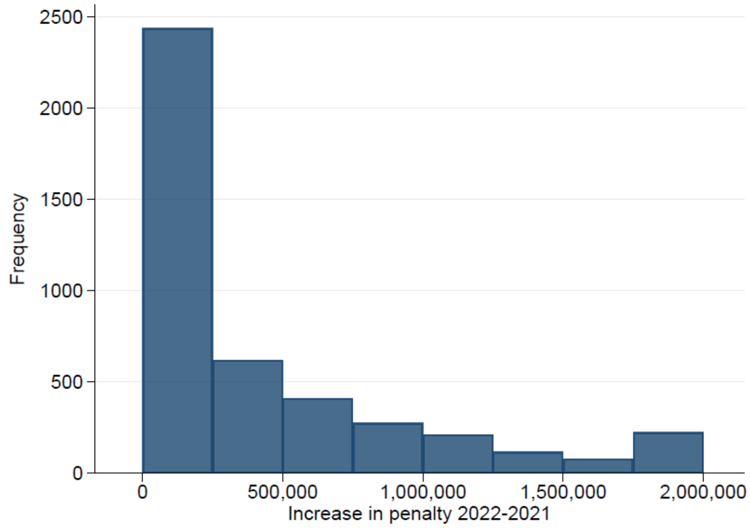

NOTES: Histogram shows the distribution of penalty increases (in dollars per year) from 2021 to 2022 across hospitals.

© 2023 Kong E et al. *JAMA Network Open.*

eFigure 3. Penalties in 2022 as a Percentage of Hospital Revenue, by Bed Group

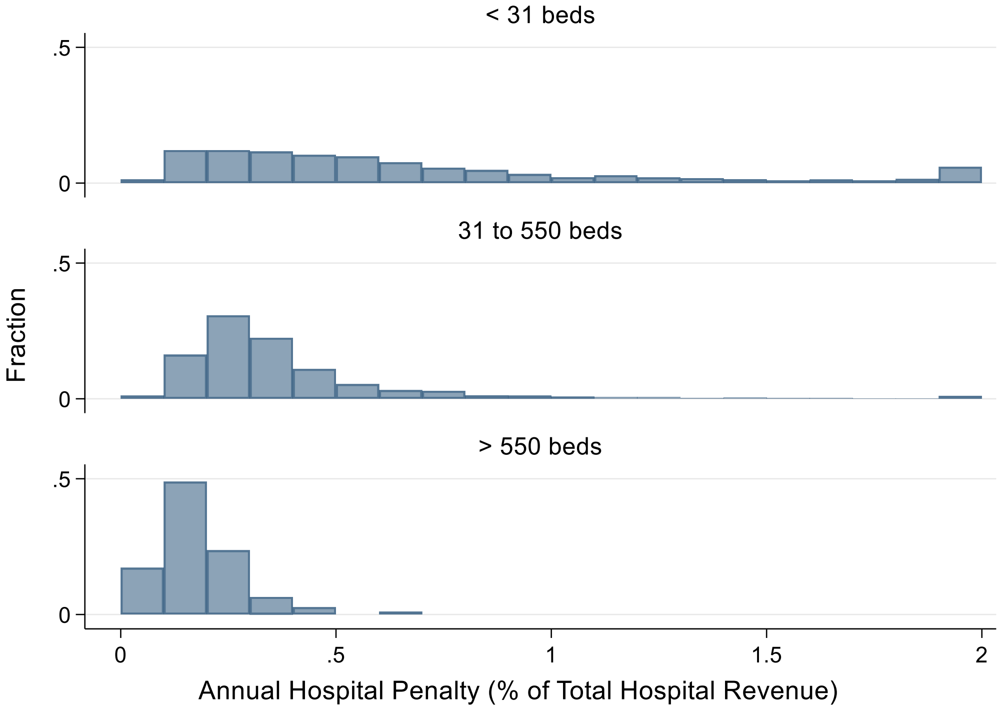

NOTES: Histograms show the distribution of annual penalties (in percentages of total annual hospital revenues), separately by bed group. Y axis is the fraction of hospitals within each bed group. Bed groups are defined in the context of the 2022 penalty schedule, using inpatient bed counts from the 2018 CMS Cost Reports data. Bed group 1 consists of 1,540 hospitals with 30 beds or fewer; bed group 2 consists of 2,651 hospitals with between 31 and 550 beds; bed group 3 consists of 186 hospitals with > 550 beds. Bed groups 1, 2, and 3 account for 35%, 61%, and 4% of the total sample of N=4,377 hospitals, respectively.

© 2023 Kong E et al. *JAMA Network Open.*

eFigure 4. Change in Penalties as a Percentage of Hospital Revenue, by Bed Group

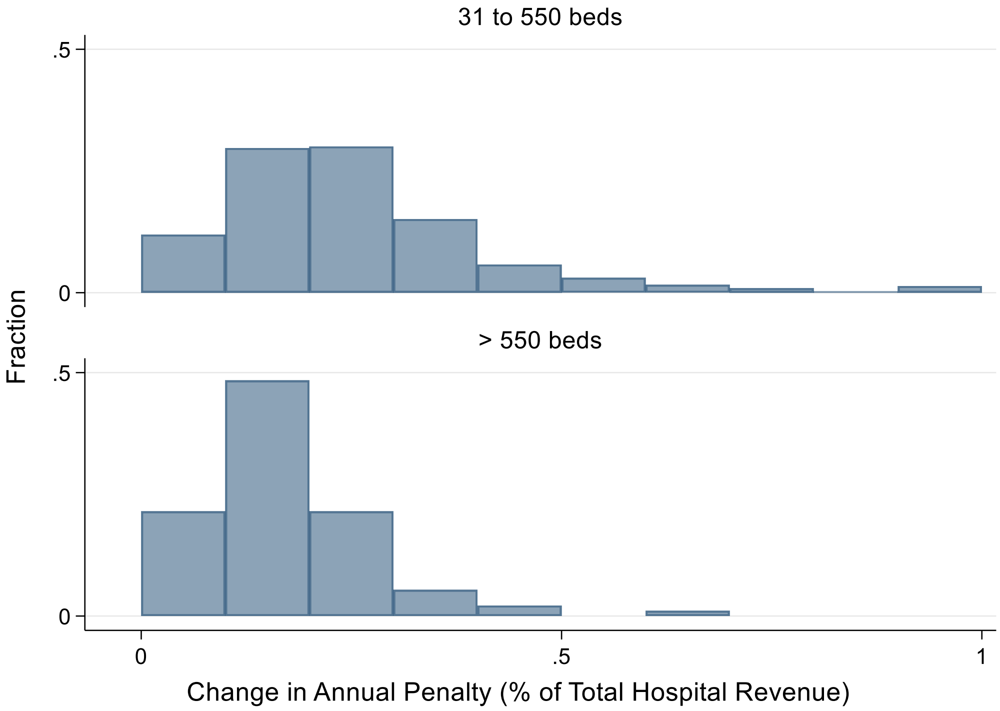

NOTES: Histograms show the distribution of change in annual penalties (in percentages of total annual hospital revenues) between 2021 and 2022, separately by bed group. The bed group with 30 and fewer beds is omitted because the change in penalties was zero.

© 2023 Kong E et al. *JAMA Network Open.*

eFigure 5. Change in Compliance as a Function of Beds (Linear X-Axis Scale)

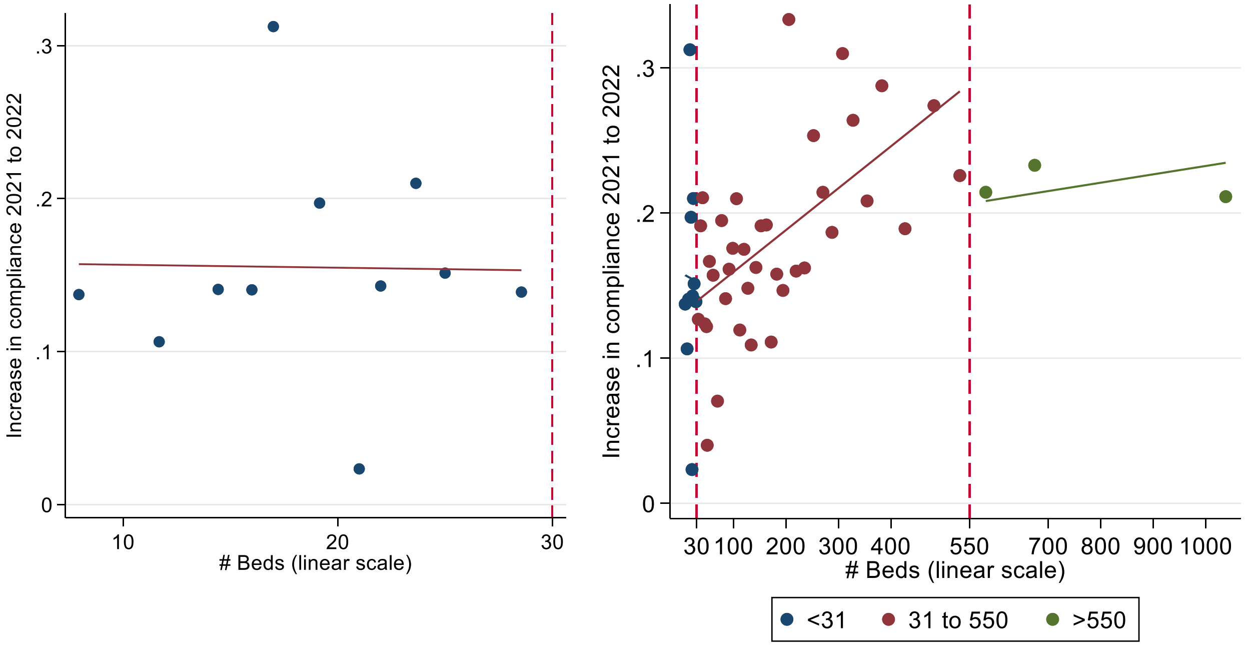

NOTES: The figure shows a binned scatterplot of changes in compliance share vs. bed count, which determines penalties. This reproduces Figure 2 in the main text, but with a linear scale for the X-axis rather than a log scale. Penalties are constant for bed counts of 0-30 and 551+, but increase linearly with bed counts between 30-550. Panel A of the figure shows a zoomed-in version of the 0-30 bed count range. Panel B of the figure shows the full range of beds on the X-axis. Figure shows N=4,377 hospitals binned into 50 separate bins with a median bin size of 77 hospitals.

© 2023 Kong E et al. *JAMA Network Open.*

eFigure 6. Negative Control Based on 2021 Compliance

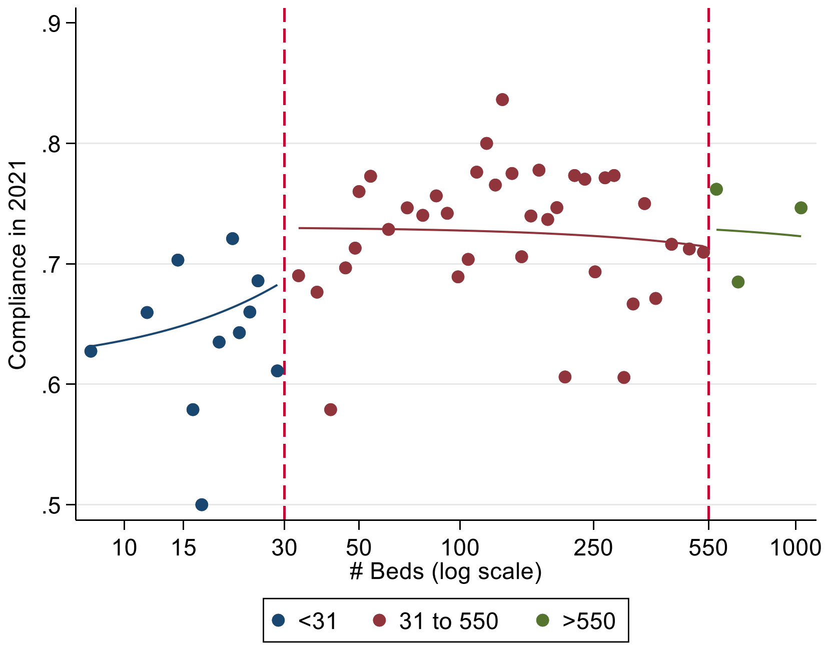

NOTES: The figure shows a binned scatterplot of 2021 compliance rates vs bed count as a negative control. Variation in 2022 penalties, corresponding to bed counts between 30 and 550, is not correlated with compliance in 2021, before the penalty change went into effect. Figure shows N=4,377 hospitals binned into 50 separate bins with a median bin size of 77 hospitals.

© 2023 Kong E et al. *JAMA Network Open.*

eTable. First Stage and Reduced Form Regressions

	**First stage** Penalty change	**Reduced form** Compliance change		**Negative control** Compliance in 2021	
Bed Group X Number of Beds					
< 31 beds	0	-0.00019		0.00248	
		(.00201)		(.00209)	
31 to 550 beds	3650	0.00029	**	-0.00003	
		(.000069)		(.000072)	
> 550 beds	0	0.00006		-0.00001	
		(.000112)		(.000117)	
Bed Group					
31 to 550 beds	-109500	-0.02812		0.11884	*
		(.0469)		(.0489)	
> 550 beds	1898000	0.01614		0.12343	
		(.104)		(.109)	
Intercept	0	0.15867	**	0.61183	**
		(.0446)		(.0464)	
Number of observations	4377	4377		4377	

NOTES: Table shows regression results for the first stage (Column 1), reduced form (Column 2) and negative control (Column 3). Standard errors in parentheses. Stars represent significance at P < 0.05 (*) or P < 0.01 (**)

© 2023 Kong E et al. *JAMA Network Open.*
