# Supplementary material for: Provision of Hospital Price Information After Increases in Financial Penalties for Failure to Comply With a US Federal Hospital Price Transparency Rule
Source: JAMA Netw Open. 2023 Jun 28;6(6):e2320694. doi: 10.1001/jamanetworkopen.2023.20694 (PMC10308252; doi:10.1001/jamanetworkopen.2023.20694)
Supplement: Supplement 2. — Data Sharing Statement [file jamanetwopen-e2320694-s002.pdf]

Data Sharing Statement

Kong. Provision of Hospital Price Information After Increases in Financial Penalties for Failure to Comply With a US Federal Hospital Price Transparency Rule. *JAMA Netw Open*. Published June 28, 2023. doi:10.1001/jamanetworkopen.2023.20694

Data
Data available: Yes
Data types: Data (not involving human participants), Data dictionary
How to access data: Data and code will be made available at https://www.yunanji.com/data
When available: With publication

Supporting Documents
Document types: Statistical/analytic code
How to access documents: Data and code will be made available at https://www.yunanji.com/data
When available: With publication

Additional Information
Who can access the data: General public.
Types of analyses: Data will be available to anyone interested in replicating our work or using our data for further academic analyses.
Mechanisms of data availability: Data will be available for download. No additional approval necessary.
